# “A very humiliating illness”: a qualitative study of patient-centered Care for Rifampicin-Resistant Tuberculosis in South Africa

**DOI:** 10.1186/s12889-019-8035-z

**Published:** 2020-01-17

**Authors:** Jennifer Furin, Marian Loveday, Sindisiwe Hlangu, Lindy Dickson-Hall, Sacha le Roux, Mark Nicol, Helen Cox

**Affiliations:** 1 Department of Global Health and Social Medicine, Harvard Medical School, 641 Huntington Ave, Boston, MA 02115 USA; 2 Health Systems Research Unit, South African Medical Research Council, Cape Town, South Africa; 3 Centre for the AIDS Programme of Research in South Africa, Nelson R Mandela School of Medicine, University of KwaZulu-Natal, Durban, South Africa; 4 Division of Medical Microbiology, Department of Pathology, University of Cape Town, Cape town, South Africa; 5 Institute of Infectious Disease and Molecular Medicine and Wellcome Centre for Infectious Diseases Research in Africa, University of Cape Town, Cape Town, South Africa; 6 Division of Infection and Immunity, School of Biomedical Sciences, University of Western Australia, Perth, Australia

**Keywords:** South Africa, Costs, Challenges, Social support, Counseling

## Abstract

**Background:**

Patient-centered care is pillar 1 of the “End TB” strategy, but little has been documented in the literature about what this means for people living with rifampicin-resistant (RR-TB). Optimizing care for such individuals requires a better understanding of the challenges they face and the support they need.

**Methods:**

A qualitative study was done among persons living with RR-TB and members of their support network. A purposive sample was selected from a larger study population and open-ended interviews were conducted using a semi-standard interview guide. Interviews were recorded and transcribed and the content analyzed using an iterative thematic analysis based in grounded theory.

**Results:**

16 participants were interviewed from three different provinces. Four distinct periods in which support was needed were identified: 1) pre-diagnosis; 2) pre-treatment; 3) treatment; and 4) post-treatment. Challenges common in all four periods included: socioeconomic issues, centralized care, and the need for better counseling at multiple levels.

**Conclusions:**

Beyond being a “very humiliating illness”, RR-TB robs people of their physical, social, economic, psychological, and emotional well-being far beyond the period when treatment is being administered. Efforts to tackle these issues are as important as new drugs and diagnostics in the fight against TB.

## Introduction

The World Health Organization (WHO) has emphasized the importance of “patient-centered care” in efforts to End Tuberculosis (TB) [1]. Defined as “providing care that is respectful of, and responsive to, individual patient preferences, needs, and values, and ensuring that patient values guide all clinical decisions,” patient-centered care is important enough to form pillar one of the WHO’s strategy to eliminate TB in the next decade [2]. While advocates prefer the term “person-centered care” so that an individual is not defined by his or her disease, [3] this attention to the unique needs of people living with TB is a welcome change in a field that has largely been dominated by a de-humanizing public health approach [4].

Despite wide-spread use of the term there is only a general definition of what person-centered care means. The End TB strategy document, for example, describes the following types of services that could be considered elements of patient-centered care: health education; improved communications (including using digital technologies); adherence support (including nutritional, financial, and transportation); psychological support; de-centralized services; and staff education [5]. There is limited documentation, however, of the specific needs and values of people living with TB, how these needs change during their illness and what kind of support people living with TB receive to address their needs [6]. Nowhere is this more apparent than in the diagnosis and treatment of rifampicin-resistant forms of TB (RR-TB), a type of TB that causes almost 600,000 people to become sick annually. [7]. Only a small percentage of people living with RR-TB are diagnosed with the disease or started on treatment [8] Those who are face an arduous treatment journey involving months of therapy with highly toxic agents that cure just over half of them [9, 10]. The diagnosis and treatment of RR-TB is fraught with difficulty, and some people living with RR-TB have described the treatment as being worse than the disease [11].

In this context, what would constitute “patient-centered care”? Studies reveal multiple challenges faced by people with RR-TB, including: economic struggles; food insecurity; discrimination and stigma; depression and anxiety; difficulty accessing therapy; hospital-based care; and side effects from treatment [12–17]. Some studies have documented that addressing these challenges by providing nutritional supplementation, conditional cash transfers, counseling, and peer support groups can result in improved TB outcomes [18–21]. Yet little is known about the specific support needs identified by people living with RR-TB themselves and their caregivers in a high-burden RR-TB context. We report a qualitative study describing the meaning of ‘patient- or people-centred care’ from the perspectives of people treated for RR-TB in South Africa and their supporters, including patients’ experiences accessing and receiving care for RR-TB from the time f symptom development through treatment completion.

## Methods

Open-ended qualitative interviews were done with 16 participants between November, 2018 and March, 2019. Eight were people treated for RR-TB and eight were persons who provided support to individuals during RR-TB treatment.

This qualitative study was part of a larger health systems study led by the University of Cape Town assessing implementation of, and access to, decentralized treatment for RR-TB in South Africa. [22] The goal of this larger study was to describe patient pathways through different levels and healthcare facilities in order to visualize the extent to which RR-TB treatment was decentralized across different geographic and health system settings. This study took place in three South African provinces: 1) Eastern Cape, which has a largely rural population and limited resources; 2) KwaZulu-Natal, which has high rates of both RR-TB and HIV and a large urban population; and 3) Western Cape, which has a mix of urban and rural populations, a lower rate of HIV co-infection than the other provinces, and more access to health care resources.

The sample for this qualitative study was selected purposively from this larger study population to ensure representation of at least two individuals from the following categories: 1) urban versus rural; 2) varied socioeconomic status (as indicted by pre-diagnosis employment) and 3) use of newer TB drugs in their treatment regimens. Support persons were identified by the original participant, except for the support people of two participants who had died. In these instances, clinic staff were asked to identify key support people.

A semi-structured interview guide (see Additional file 1) was used to ask about challenges with RR-TB diagnosis and care, sources of support during care, treatment preferences, suggestions for improving care, and any additional comments or concerns of their treatment experience. Given the changing landscape of RR-TB treatment, more structured questions were asked about treatment preferences, including whether a person preferred all-oral therapy and shorter therapy duration, and how these preferences would change if the chance of cure differed with the options offered. Interviews were carried out by two interviewers (SH, ML) in the preferred language of the participant (isiZulu, isiXhosa, Afrikaans or English, with translation if needed) and recorded. All recordings were transcribed directly into English. Transcripts were coded, entered manually into Microsoft Excel and analyzed to generate themes, assess content and identify patterns. A total of 16 participants were interviewed: 12 included pairs of patients and supporters; two were supporters of patients who died; and two were patients who declined to name a supporter to be interviewed.

**Fig. 1:**
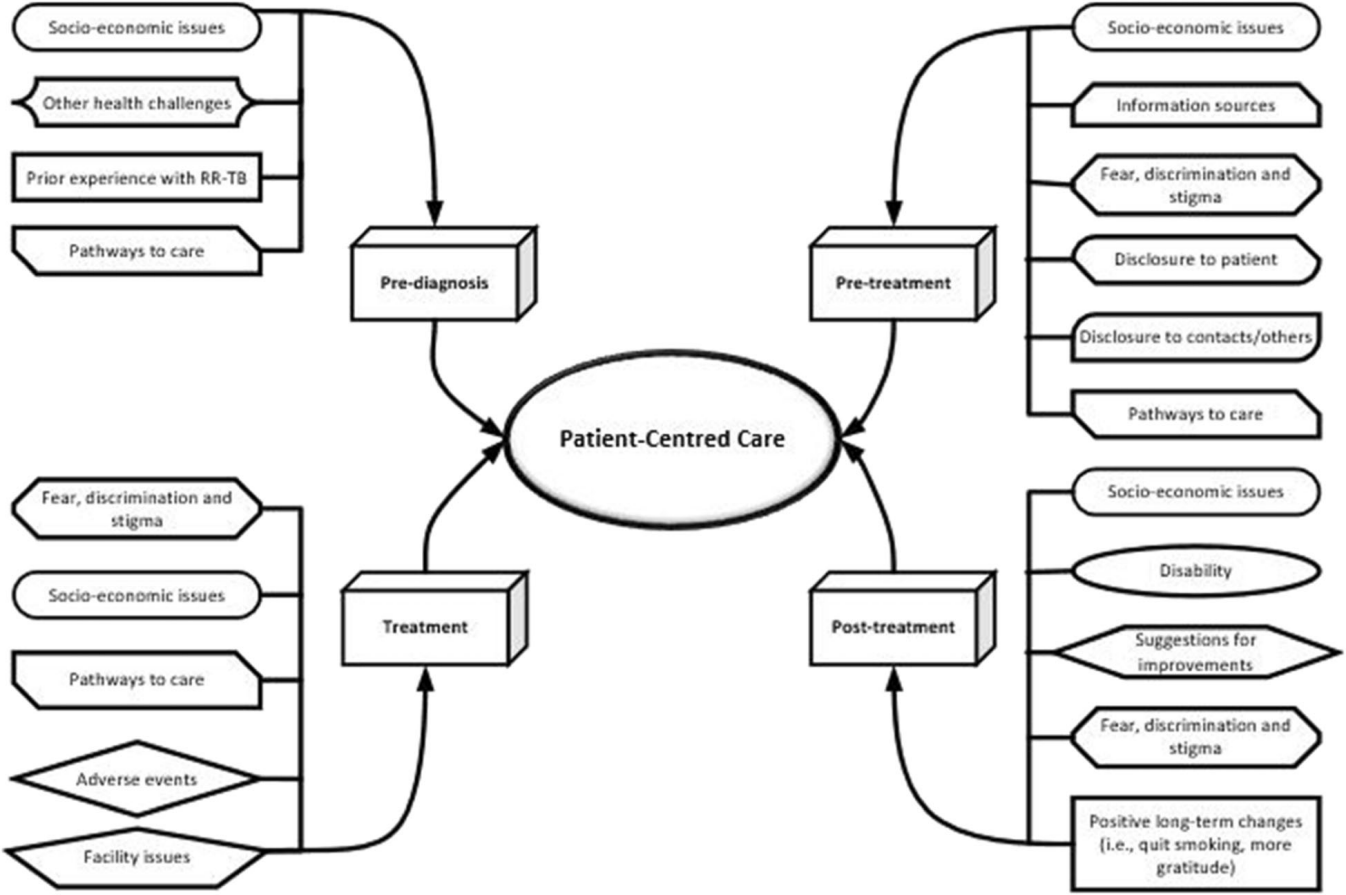
Thematic network analysis of Patient –Centered Care

A thematic network analysis was used to analyze the data from the transcripts (Fig. 1) [23, 24]. The analysis was iterative in that: interviews were transcribed immediately after the interview, transcripts were reviewed by the team, and the interview guide updated to reflect new information (i.e. a participant mentioned a positive result from having RR-TB was that he stopped smoking, so participants in subsequent interviewers were then specifically asked about positive aspects of having RR-TB). After an initial review of the data, a coding system was developed by one study team member (JF), verified/modified by another (ML), and the first 16 interviews were analyzed. Discrepancies were resolved via discussion and there was agreement among all study team members on the final analytic framework used. Interviews were halted after the initial 16 participants since inductive thematic saturation had been reached (determined by two team members, JF and ML), [25] as no new codes or themes were emerging in the dataset [26].

### Ethics

This study was approved by the Human Research Ethics Committee at the University of Cape Town (350/2016). The study was explained to all participants and written consent for participation and recording was obtained.

### Terminology

The term “patient” describes the person who was treated for RR-TB. The term “supporter” describes the person or persons who were identified by the patient as their key source of support. The term “participant” includes both patients and supporters.

## Results

Sixteen interviews were conducted pertaining to the care and treatment of 10 patients (Table 1). Eight patients were interviewed and they came from urban settings (*N* = 4), rural settings (*N* = 3) and a correctional facility (*N* = 1). Two of the patients were unemployed and six were employed prior to being diagnosed with RR-TB. Two of the patients received the new drug bedaquiline during treatment. In order to ensure that the experience of patients who had unsuccessful outcomes were documented, two supporters of patients who died were also included. Four distinct time periods were identified by patients and supporters in which they faced unique challenges requiring support. Each of these is described in detail below, including the key activities that had to be accomplished, challenges faced by the patient and his or her supporters, and supporting factors throughout each period of treatment (Table 2).

**Table 1:** Characteristics of participants and description of interviews conducted

Number	Patients interviewed	Gender (patient)	Age of patient (years)	Rural, Urban, or Correctional Facility	Employed at the time of diagnosis (yes or no)	Number of Supporters Interviewed	Gender (supporter)
1	1	F	36	Urban	No	0	
2	1	M	30	Urban	Yes	1	F
3	1	M	36	Rural	Yes	1	F
4	1	M	22	Urban	Yes	0	
5	1	M	53	Rural	Yes	1	F
6	0	F	36			1	F
7	1	M	48	Correctional Facility	No	0	
8	1	F	24	Urban	Yes	3	2 M, 1 F
9	1	M	29			0	
10	0	F	27	Rural	Yes	1	F
Total interviews	8					8

**Table 2:** Challenges and Supporting Factors which Emerged in the Four Phases of RR-TB Treatment

	Pre-diagnosis: Patient was symptomatic but had not yet been diagnosed with RR-TB	Pre-treatment: Patient had been diagnosed with RR-TB but had not yet been started on treatment	Treatment: Patient was prescribed and/or taking treatment for his or her RR-TB	Post-treatment: RR-TB treatment was complete but the patient was still managing consequences of having survived RR-TB.
Challenges				
		A very confusing and difficult time. Multiple challenges to negotiate		
Physical challenges	Physical symptoms disrupted normal activities of daily living		Adverse events experienced by all patients. These varied from those that impacted significantly on patients’ lives to being tolerable.	Permanent disability due to treatment
	Additional health challenges eg. pregnancy and co-morbidities		Pill burden difficult to tolerate	
			Patients weak and inadequate physical support from hospital staff to bath etc.	
			Adverse events not always addressed in a timely fashion	
Health system challenges	Long waiting times and long queues at all health facilities.			
	Health system complicated and challenging to negotiate	Multiple care providers at different facilities: Co-ordination and communication between them sub-optimal	After discharge from hospital, due to poor communication there was inadequate care at outpatient facilities.	Inadequate information on adverse events and possible permanent disabilities
	Multiple visits prior to diagnosis	Accessing RR-TB services necessitates long distance travel. RR-TB patients stigmatised, so that travel is discriminatory and frightening	Shorter regimen preferable, but longer regimen preferable if chance of cure increased and pill burden decreased	
Economic challenges	Loss of income from not working. Additional expenditure of the cost of transport to health facilities			Due to permanent disability unable to continue working - severe economic impact on the household
			Confusion regarding access to disability grants during treatment.	
Emotional and psychological challenges		Receiving news of diagnosis and the implications of this diagnosis	Loss of identity	Sense of loss and anger with permanent disability. No longer the same person
		Anxiety and concern about infecting others		Anxious about becoming ill with RR-TB again
Social challenges	Unable to continue with household responsibilities eg. child-minding, cleaning	Disclosure – implications and fear of stigma and discrimination	Hospitalisation – someone else needed to take over family and household responsibilities	Inadequate community awareness and understanding of TB and its transmission
		Stigma affected whole family, including at work	Social isolation during hospitalisation due to transport costs for family to visit patient	
		Some sources of support rejected the patient on hearing their diagnosis	Continued stigma and discrimination	
		Disruption of family relationships		
		Masks – a visual sign of stigma and discrimination		
Supporting factors				
	A previous experience of a family member who had had RR-TB	Importance of nurses: Main providers of information, care and support	Nurses identified as the most important source of support and information both in hospital and after discharge	Need for support continued after treatment completion
	Relocation back to family for support		Religious faith and the support of religious leaders important for some patients.	
	Physical support with activities of daily living difficult (ADL)			
	Support with household responsibilities eg. child-minding as visiting facilities took time			
	Emotional support and encouragement by family member to keep going to health facilities			

### Pre-diagnosis period (usually lasting several weeks to months)

The hallmark of the pre-diagnosis period was that the patient was physically sick but did not know the underlying cause of their symptoms. Key tasks which had to be accomplished were managing symptoms and finding out the diagnosis, whilst they continued to provide financially for their families or played another key family role (such as child care).

All patients reported feeling physically sick and visiting at least two healthcare facilities prior to their RR-TB diagnosis. Some were initially told they did not have TB. During this period, some patients had to leave their place of residence and move back home to be with family members who could provide physical care or help with housing, money, food, and/or child care. All six of the eight patients who were working when they developed symptoms altered their jobs or duties which led to a reduced income. This economic loss was compounded by incurring additional expenses whilst trying to obtain a diagnosis. Two patients reported providing care for other family members who had RR-TB, a largely negative experience:

*“It could be that I got [RR-TB] from my family member because I was the one that was taking care of her … I was the one to transport her with a wheel barrow to the ambulance.”* (male, age 30)

Only a few patients had support during this period. This was usually from family members/spouses (almost always females) and included encouragement, emotional support, childcare, and in some cases, monetary support. Supporters identified at this time remained the main support people throughout the four periods identified in the study.

### Pre-treatment period (usually lasting several days to weeks)

The pre-treatment period was a time of great uncertainty for patients and supporters. Key tasks during this time were: processing their RR-TB diagnosis; disclosing their RR-TB status to others and encouraging them to be checked for RR-TB; obtaining both general information about RR-TB as well as the immediate next steps for treatment; getting to a treatment center to start treatment; and managing the loss of normal roles and social identities.

In terms of disclosure of the diagnosis, most patients felt this was done in a sympathetic way either in person or telephonically by the nurse at the diagnosing clinic. However, some participants felt the nurses did not have adequate information to share with them and others did not have a positive experience.

*“ … they did not explain very clearly what was happening. I had the paper with me and then they called me to check the paper. They then said come here and put a mask on me. They told me to sit outside and wait there. They did not treat me well.”* (male, age 30).

Others reported that the nurses interacted in a kind manner, but their messages contained information meant to evoke fear, using terms like “most dangerous” to describe RR-TB and emphasizing what would happen to the patients if they did not take their treatment.

“*She was very friendly. She had a poster that she showed me about the consequences of not taking my treatment”* (male, age 29).

One of the most stressful patient tasks was disclosing their diagnosis to family members while at the same time encouraging family members to get tested for TB. This task evoked a great deal of guilt and stress for which little support was provided by the healthcare system.

*“ She even told me that I have to tell them at home to come and check because maybe I might have spread it at home.”* (male, age 30)

The task of sharing their diagnosis with family members while also telling them that they may have been infected led to feelings of guilt and shame among almost all patients, and resulted in discrimination from family members. Some reported losing support they had received during the pre-diagnosis period and some were made scapegoats for other family problems, including one family that blamed the patient’s disclosure as the reason his nephew failed to pass his school exams.

Another significant task for patients and their supporters during the pre-treatment period was being admitted to the treating hospital for initiation of therapy. All treatment initiation sites were located outside of the patient’s home community. Patients and supporters uniformly described transportation challenges. In one extreme case, reported by a supporter, the patient was told he needed to be admitted to his region’s RR-TB hospital, located far from the diagnosing clinic, and instructed to wait for an ambulance to take him there. The ambulance arrived at midnight.

*“When the ambulance came, the ambulance driver said that he was knocking-off and he was not going to [the regional RR-TB hospital] so that is when he took the patient to [the tertiary hospital an hour and a half away].”* (female supporter of female patient age 36 at time of death).

Transport challenges led to some patients realizing the implications of their disease.

*“There were three of us in this ambulance but they told me to sit in the far corner of the ambulance. I said to myself this means that I am dead*.” (male, age 36).

In the RR-TB treatment facility, however, many felt they were provided with more information and felt more welcome. Information was almost always provided by nurses at the receiving hospital.

*“As I arrived the nurses came outside and met me and they asked me what was the matter. I said I was sick and they told me that I was not sick. I noticed as I entered the ward that there were other sick people there and I didn’t seem that sick. The male nurse told me that this was going to be my home for the next three to four months”* (male, 36 years)

All the participants reported socioeconomic struggles during this pre-treatment period, both in terms of lost work and normal activities that had to be taken over by others.

Almost all socioeconomic, emotional/psychological, and social support provided was from family members and almost always from females. Of note, supporters also reported that they themselves needed emotional/psychological help as well.

### Treatment period (usually lasting 12 to 36 months)

The treatment period is often the time in the therapeutic journey of a person living with RR-TB that receives the most attention—likely because it lasts for the longest duration. Key challenges negotiated by patients during this period were: initiating treatment; dealing with adverse events; adhering to treatment; moving from the hospital back to their home communities; and managing the prolonged loss of their normal roles and identities.

Most of the participants felt the hospitals had provided good care, although there were some notable exceptions. Again, nurses were identified as the primary sources of information and support.

*“ … they were always coming on time when it was time for me to take my pills, they would even help me to take pills, I would also notice that if ever I was not feeling well, they would notice and they would ask me what is it that is making me feel down. They took good care of me but not all of them because people are not the same*.” (male, age 22).

However, not all patients felt support from the nurses. One patient who experienced multiple side effects and had a difficult treatment journey noted:

*“Yes, the nurses would just be there to get paid. They are just doing their work and working to get paid … There is no love in the hospital. The sisters and the nurses there, they tell you straight if you don't take your pills you will die.”* (male age 53)

Distance to the hospital made it hard for supporters to visit, as did the practice of not allowing family to visit patients on the wards. Deprived of their usual social support, patients identified new sources of assistance including other patients hospitalized for RR-TB treatment, cleaning staff, religious leaders, family members of other patients who were visiting, and prison wardens. This assistance varied from physical support with washing/eating, educational support, to emotional support.

“ *… I met a cleaning lady that told me that the only thing that you shouldn't do in that hospital is to get sick to the point where you lay in bed and you can't do anything for yourself because then you are going to die.” (female, age 24)*

*“The guy [s] always gave [me] hope saying that one day we will be out of this place, we came here in wheel chairs.”* (male, age 30)

*“ … the pastor also feeds them; the reason is that sometimes we come to visit a church member but when we are here, we don’t choose. We help everyone.”* (female supporter of male)

On discharge from hospital patients had to re-establish support networks within the community, which was challenging at times. Sometimes male family members provided short-term assistance with material needs, including the provision of housing or money. Again, nurses were identified as a primary source of support during this transition period. Much of this had to do with the direct physical care they provided during ongoing outpatient treatment.

*“The person giving the injection was kind and worked fast- they didn’t want me to wait long. At the end when I was gaining weight he told me that I would get better if I complete the treatment”* (female, age 36)

Every patient who participated in the study experienced adverse events. Some of these were mild and tolerable, whereas others had a significant impact on the life of the patient. Injection site pain and hearing loss were described by many patients as were nausea, vomiting and loss of appetite. Two patients reported memory loss and bad dreams. Two male patients reported impotency which was troubling to them. On discharge, one patient developed hallucinations, ran away from home and was found in a psychotic state. As his wife reported:

*“He ran into the bush... He was found on the third day at night. He was trying to cross the road but he was just covered with a blanket. He was naked. He had a wound on his arm so we don't know whether he fell down or whether he was hit by a car or what happened.”* (female, supporter of male patient age 53).

One participant reported that the treatment caused more problems for her than the RR-TB:

*“You have to take all 30 pills and finish. For me, I felt that I was not sick. I literally got sick when I started taking the pills*.” (female, age 24).

Patients reported that they adhered to treatment despite the many challenges, largely because they feared dying or infecting others. Many reported witnessing other patients in the hospital die and did not want that to happen to them.

*“There was a lot who died in front of me for not taking their pills.”* (male, age 22).

In terms of treatment preferences, several patients identified the injectable as one of the more challenging medications. However, the high pill burden was mentioned as equally challenging by others. As the supporter of a patient who died said:

*“I think it was the pills. It was a lot of pills. It was maybe more than eighteen pills. It was too much*.” (female supporter of female who died at age 27).

When asked if they would prefer a shorter (9 month) regimen or a longer (24 months) regimen, participants uniformly replied that they would prefer a shorter regimen. However, when asked for their preference if the shorter regimen did not cure as many patients as the longer one, most participants stated they preferred a longer regimen with a higher chance of cure:

*“I will go for two years. What if I am the one that will not be healed? No way!” (male age 36 years).*

While most patients stated they would prefer an injectable-free regimen, some said they would have the injectable if this reduced the pill-burden:

*“I would take the pills and the injections but only if the pills are less.”* (female, age 24)

Participants continued to experience stigma and discrimination, which had a major impact on their social identities:

*“It was actually very humiliating for me because you have to sit with a mask on your face at the clinic and people are looking at you and nobody wants to come near to you. I heard someone by the clinic saying if they are walking around like that,[you] have dangerous viruses and things … It is a very humiliating illness*.” (female age 24).

All patients lost the ability to contribute economically during treatment, and many had to have assistance with their normal social roles, for example as mother. Some of the patients received a disability grant—and mentioned that social workers were a great source of support. Two patients, however, were told they could not apply for a disability grant as they did not qualify and one patient was told he would face legal consequences and “be arrested” if he applied, as the social worker felt he could still work. One patient reported receiving one food package from the Red Cross, but no other sources of nutritional support were described.

### Post-treatment period (ongoing)

This period was defined as the time when RR-TB treatment was completed but when patients were still wrestling with the impact of RR-TB in their lives. Key tasks were: managing permanent side effects; re-integrating back into society/roles; managing concerns about recurrent RR-TB disease; and managing overall health.

Four of the patients who were treated for RR-TB developed permanent disabilities as a result of their treatment, usually injectable-related hearing loss. These patients and their families felt a profound sense of loss and anger:

*“These nurses didn’t even explain to me about the injection. I discovered it when I was there, I must also get an injection. They never even told me that my ears would be closed and they never told me that maybe I will end up no longer being the person that I used to be*.” (male, age 53).

Some participants did describe longer-term benefits from their RR-TB diagnosis. Two reported they had stopped smoking during treatment, one that he had stopped drinking alcohol during treatment and one reported a sense of thankfulness:

*“It did change some things. I stopped smoking … I somehow had no choice because I could not mix cigarettes with my treatment”* (male, age 30)

All participants were asked what could be done to improve the RR-TB services. Many recommended facility-based changes to reduce waiting times at facilities, shorten queues, and/or provide more support with daily activities to weak hospitalized patients. One participant specifically mentioned the need for more support staff to feed and bathe patients too weak to do so themselves. Others mentioned changing treatment regimens to make them easier to take by reducing the daily pill-burden and the side effects.

Participants were clear about what they would tell other people suffering from RR-TB. Most stated they would emphasize the importance of “following all the rules” and “taking all the medicines.”

Of note, no new sources of support were recruited or acquired during the post-treatment period by patients. However, some of the participants reported feeling more closely linked with their supporters. In one illustrative example, one of the cured patients got the phrase “Stay Strong” tattooed on her body. Her father was also going to get the same thing tattooed on his arm in solidarity.

*“I want to tell the story why I have ‘Stay Strong’ written on my body.”* (male supporter of female patient, age 24).

## Discussion

This qualitative study identified numerous challenges faced by people living with RR-TB that occurred in four distinct time periods. While many of these challenges were unique to some patients and supporters, others were cross cutting, including socioeconomic issues; problems due to centralized care; the need for better counseling for patients and supporters; and more interaction with clinical staff. While some of these challenges are hinted at in larger and more general concepts of “person-centered care”, [27] our findings show many of them are not addressed by National TB Programs (NTPs) or existing health systems.

The interviews highlighted constant economic challenges from the time symptoms developed through the post-treatment period, usually caused by lost work, new expenses (especially travel and transport) and the need to recruit additional help to take care of tasks normally done by the patient (e.g., childcare, household chores) These findings reinforce previous studies highlighting the “catastrophic” costs associated with TB and RR-TB treatment, even when such treatment is provided free of charge [28], [29]. These socioeconomic challenges highlight the need not only for social protection strategies to mitigate the cost of illness and to prevent individuals with TB from being forced further into poverty, but also for direct socioeconomic support to enhance the chances of treatment success. [30] In South Africa, although all patients with RR-TB are eligible for disability grants for the duration of treatment, [31] not all were advised to apply and assisted with their applications. These data suggest that skilled social workers have a major role to play in the treatment of RR-TB. However, previous studies have suggested that access to such grants is often delayed and does not provide economic support in the crucial first few months of treatment. [32] Incorporating social protection strategies directly into NTPs may help to eliminate catastrophic costs, but it is unlikely that this will be accomplished by the target date of 2020. [33] Social protection should be a central component of a person-centered approach to care. In fact, studies have found that economic support linked with counseling improves treatment adherence and the chance of cure. [34]

The results also highlight the numerous challenges that result from centralized treatment services. Patients treated within decentralized models of care have treatment outcomes that are similar compared with centralized care, and health systems costs are lower with decentralized services [35–37]. Implementation of decentralized care, however, has been limited by concerns around the provision of “specialized” treatment in decentralized settings. This study shows that even though many participants reported positive in-hospital experiences, centralized care was often not person-centered. People living with RR-TB were removed from their existing sources of support and found the transfers in and out of communities to be times of greatest stigma and discrimination. Furthermore, the economic burden on patients and households during hospitalization was significant due to the loss of income and the extra transportation expenses incurred.

Finally, this study showed that there is a dire need for improved education and counseling for patients, their supporters, and the health care staff who provide care. In terms of health care staff, nurses were mentioned by all patients as the most significant sources of support. As such, nursing staff appear to be the focal points for delivering person-centered care. This crucial role must be recognized, professionalized and supported by other members of the health care team. Nurses at all levels should be provided with more in-depth training on RR-TB treatment, helping patients manage disclosure, how to minimize stigma and discrimination, and how to help patients transition through the different phases described in this paper. Reinforcement of more positive and hopeful messaging should also be emphasized. Ancillary staff were also reported as sources of information and support. NTPs should consider more formal training for, recognition of, and utilization of these individuals with appropriate compensation. Some examples where ancillary staff could be utilized include peer support groups, peer networks, and inclusion of these individuals as “TB champions”. Of note, the only role mentioned for doctors by most participants was deciding about the initial treatment regimen.

People living with RR-TB need more counseling and support from the health care system. One area that needs to be urgently addressed is disclosure counseling (especially to family members)—which should be built into contact tracing programs. In addition, in the post-treatment period, after being discharged as ‘successfully treated’, people still had significant medical needs. Some of these could potentially be managed through improved primary care services provided as part of universal health care. [38] Many participants described the donning of a mask as an activity that was associated with shame and stigma. While the risk of transmission may be significant in the pre-diagnosis, pre-treatment and very early treatment periods, risk likely declines rapidly with effective treatment. [39] Hence, it is important that evidence-based (and not fear-based) infection control be practiced throughout the lengthy treatment. For example, more universal mask wearing within the health care system, could help decrease both stigma and TB transmission. [40] There have been some positive experiences in parts of South Africa where primary care clinic attendees all wear paper masks as part of administrative infection control measures. [41]

Informed decision-making is one of the tenets of patient-centered care. [42] When asked about treatment preferences patients reported preferring shorter and injectable-free regimens, but they leaned toward regimens with the highest rate of cure. In addition, several also noted that the pill burden and side effects were challenging and they might be willing to take an injection if it meant fewer pills. While most patients in this study indicated they would defer to the doctor for treatment decisions, they did want to be informed about the risks and benefits of treatment and have their preferences considered and discussed when deciding on the therapeutic approach. This is rarely done in the care of people with RR-TB and must be a central component of RR-TB treatment moving forward. [43]

In terms of patient supporters outside of the health system female family members provided almost all support given to patients. As such, these women are vulnerable to worsening poverty, [44] and becoming sick from RR-TB themselves. [45] Their needs should also be addressed as a core part of patient-centered care. It was also notable that the support person in the pre-diagnosis period usually remained as core support throughout the treatment journey. These findings suggest that NTPs should identify key patient supporters early in the diagnostic and treatment process, provide counseling, education and, if possible, support to these family members, and find ways to involve them in all aspects of treatment.

There are several limitations to this study. First, the sample is small, and although purposive sampling was done, these findings may not be generalized to other populations. Second, participants were all asked about their treatment experiences retrospectively, and it may be more beneficial to follow people throughout the treatment journey and document their experiences prospectively. Finally, although we interviewed the supporters of two patients who had died, and one patient who initially was unable to adhere to treatment, the remaining patients were successfully treated. Individuals with poor treatment outcomes including those who do not complete the full course of treatment may provide different perspectives.

## Conclusion

In spite of these limitations, the study has important ramifications for TB policy and practice. Many in the larger TB community have embraced the concept of “patient-centered care” but few efforts have been made to implement such services. If this term is to move beyond popular cosmetic use—which it must if we are serious about eliminating TB—more work is needed to ensure that the unique challenges faced by each individual with RR-TB and their support networks are addressed. There also needs to be accountability when such needs are not met. The way the global community responds to the failure to eliminate TB-associated catastrophic patient costs will indicate how serious we are about putting substance behind this popular slogan. As this study has shown, beyond being a “very humiliating illness,” RR-TB robs people of their physical, social, economic, psychological, and emotional well-being far beyond the period when treatment is being administered. Efforts to tackle these issues are as important as new drugs and diagnostics in the fight against TB.

## Supplementary information

**Additional file 1:** Study interview guide: contains the questions used to guide the open-ended interviews.

## Data Availability

All data are available upon request from the corresponding author.

## Abbreviations

NTP: National Tuberculosis Program
RR: Rifampicin-resistant
TB: Tuberculosis
