# Supplementary material for: “A very humiliating illness”: a qualitative study of patient-centered Care for Rifampicin-Resistant Tuberculosis in South Africa
Source: BMC Public Health. 2020 Jan 17;20:76. doi: 10.1186/s12889-019-8035-z (PMC6969445; doi:10.1186/s12889-019-8035-z)
Supplement: Supplementary file 1 — Additional file 1: Study interview guide: contains the questions used to guide the open-ended interviews. [file 12889_2019_8035_MOESM1_ESM.docx]

**Revised Open-Ended Interview Guide July 29, 2018**

The purpose of this interview is to learn about your treatment experience when you were receiving care for drug-resistant tuberculosis (DR-TB). I am interested in hearing about what it was like for you from the time you first started feeling sick up until today, and I am especially interested in hearing about what was difficult for you and what was helpful for you throughout your treatment journey. There is great interest in improving the care provided to individuals with DR-TB, but in order to do so, there is a need to know what it has been like for people such as yourself. So thank you for making the time to share your treatment experience with me.

1. To get started, why don’t you tell me a little bit about yourself?

2. Tell me about when you first became sick and how you were given a diagnosis of DR-TB.

Probes: What symptoms did you have? Where did you go to seek medical care? Did you have to travel far to get medical care? Who told you that you had DR-TB? How did they tell you? How did you feel about this diagnosis? How did people treat you when they found out? Was anyone helpful and supportive? Was anyone rough or uncaring? Did you have to spend money during the diagnosis? Did you have to miss work?

3. Tell me about what the treatment was like for you.

Probes: Where did you get treatment? Did you have to travel to receive your medical care? What was the most difficult part of treatment? Was anyone helpful and supportive? Was anyone rough or uncaring? What kind of side effects did you have? Were you informed about the side effects? Did you have to spend any money during the treatment? Did you have to miss work?

4. If you were in charge of the TB program, what would you do differently/change to make it easier for patients?

Probes: Ask “what else” and “why” until no further answers elicited. What advice would you give to other people with DR-TB?

5. How has your experience with DR-TB impacted your life now?

Probes: How is the quality of your life now compared to before you started treatment? Have you had any long-term consequences? Are there things you cannot do now that you used to be able to do?

6. There is a lot of research being done in DR-TB now to try and find better drugs and treatment regimens. The hope is to be able to treat DR-TB with better drugs that have fewer side effects and also to be able to give treatment for a shorter period of time.

If you had a choice between receiving treatment for 24 months or 9 months, which would you prefer and why?

If the 24 month treatment cured 10 out of 10 patients but the 9 month regimen only cured 9 out of 10 patients which would you prefer and why?

If you had a choice between receiving treatment with just pills/tablets or receiving treatment with pills/tablets and an injection, which would you prefer and why?

If the treatment with pills/tablets and injection cured 10 out of 10 patients and the treatment with just pills only cured 9 out of 10 patients, which would you prefer and why?

Would you want your doctor to discuss these different options with you, or would you prefer the doctor just made the choice he or she thought was best? Tell me more about why.

7. Is there anything else you would like to tell me about your experience with DR-TB that you think it is important for people to know about?

8. You mentioned that X person was very helpful to you during your treatment journey with DR-TB. Would you mind if we contacted him/her to talk about how he/she felt about your treatment experience?
